# Deciphering the role of LncRNA in alcoholic liver disease: Mechanisms and therapeutic potential

**DOI:** 10.1097/MD.0000000000040378

**Published:** 2024-11-08

**Authors:** Lin Zhang, Rongqi Wang, Yuemin Nan, Lingbo Kong

**Affiliations:** a Department of Traditional and Western Medical Hepatology, The Third Hospital of Hebei Medical University, Shijiazhuang, China.

**Keywords:** alcoholic liver disease, diagnostic markers, long non-coding RNA, mechanism of action

## Abstract

Alcoholic liver disease (ALD) is a spectrum of liver damage caused by chronic alcohol consumption. The disease progresses in stages, starting with simple fatty liver, progressing to alcoholic hepatitis and potentially leading to fibrosis and cirrhosis. The pathophysiology of ALD is complex and involves several cellular and molecular mechanisms. Recent research has highlighted the role of long non-coding RNAs (LncRNAs) as critical regulators in the development and progression of ALD. This article reviews the current understanding of LncRNAs in ALD, focusing on their functions in key pathological processes and their potential as diagnostic markers and therapeutic targets.

## 1. Introduction

Alcoholic liver disease (ALD) is a growing public health problem worldwide, and heavy alcohol consumption is an important risk factor for the development of the disease.^[1]^ Its manifestations are diverse, ranging from mild fatty liver to more severe alcoholic hepatitis, hepatic fibrosis and the potential for further progression to cirrhosis and hepatocellular carcinoma.^[2,3]^ Severe short-term alcohol abuse can lead to acute severe alcoholic hepatitis, acute liver failure and even death.^[4,5]^ The pathogenesis of ALD is not fully understood and its treatment remains a major challenge. Therefore, research into the pathogenesis and therapeutic targets of ALD is of great clinical importance.

Long non-coding RNA (LncRNA), which make up 80% to 90% of all non-coding RNAs, are a class of non-coding sequences with a transcript length of more than 200 nt.^[6]^ They rarely encode proteins or are unable to do so because they lack effective open reading frames.^[7]^ Most LncRNAs are transcribed by RNA polymerase II or other RNA polymerases and undergo a splicing process similar to that of mRNAs via 5-syntactic end-capping (7-methylguanosine [m7G]) and a polyadenylation form at the 3m end. LncRNAs are abundant and have a wide range of biological roles, performing specific functions depending on their intracellular localization.^[8]^ They are mainly involved in the regulation of gene expression, including DNA replication and transcription, protein translation and epigenetic aspects.^[9]^ Nuclear localized LncRNAs can act as transcription factors or chromatin modifying complexes.^[10]^ In contrast, cytoplasmic LncRNAs can directly regulate mRNA stabilization or act as endogenous competing RNAs that serve to regulate functional proteins.^[11]^

Since LncRNAs influence the development of ALD through a variety of mechanisms, it is important to study the role and mechanism of LncRNAs and explore their potential as diagnostic and therapeutic targets.

## 2. Mechanisms of LncRNA action in alcoholic liver disease

**Figure 1.**
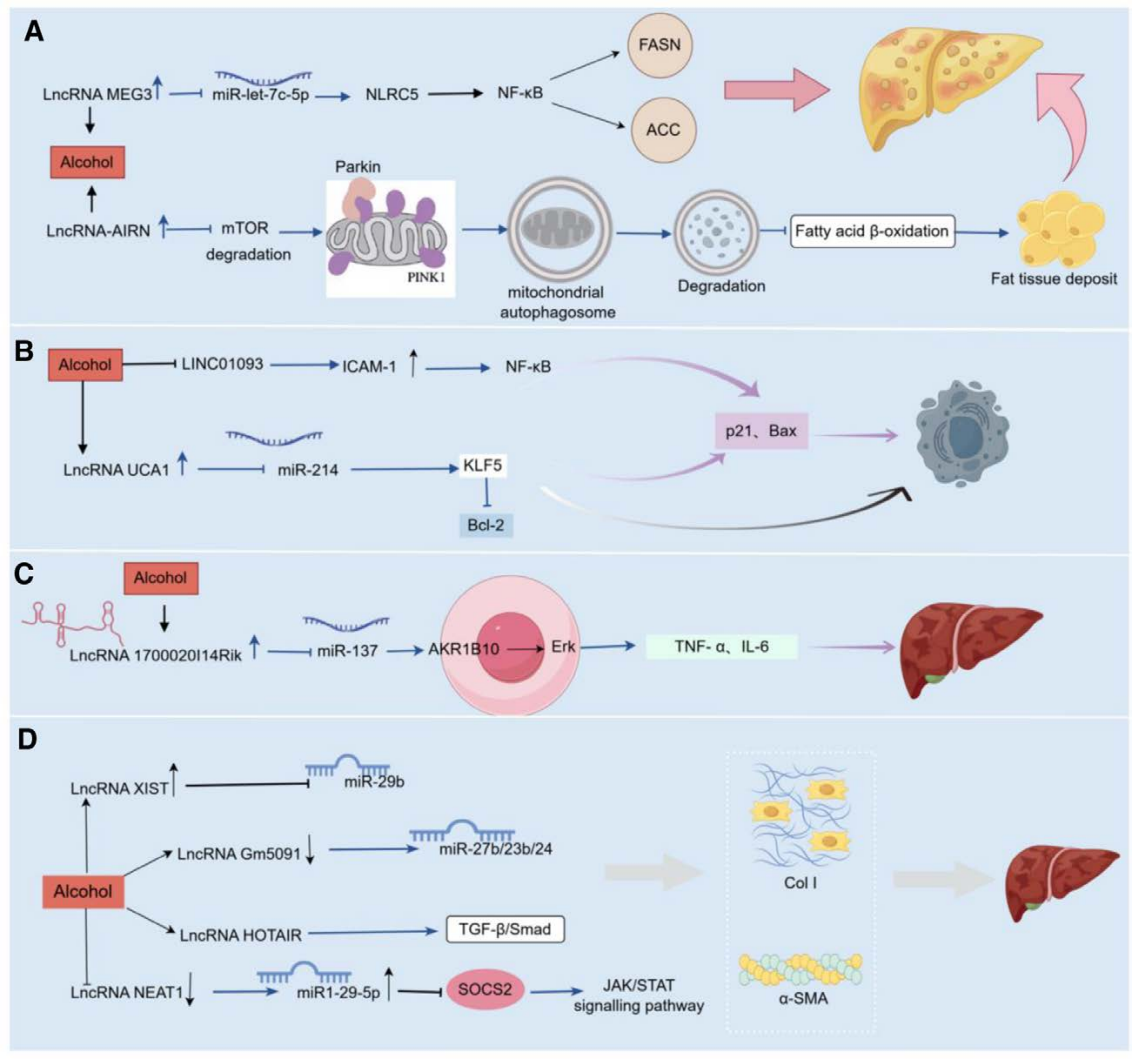
The overall process of this study. (A) LncRNA-mediated lipid dysregulation in alcoholic fatty liver. (B) LncRNA-driven apoptosis in alcohol-induced liver damage. (C) LncRNA modulation of inflammatory pathways in alcoholic hepatitis. (D) LncRNA-regulated fibrogenesis in alcoholic liver fibrosis. AKR1B10 = aldosterone reductase family 1B10, HSCs = hepatic stellate cells, ICAM-1 = intercellular cell adhesion molecule-1, NLRC5 = nucleotide binding oligomerization structure domain-like receptor C5, NLR family CARD domain containing 5, PINK1 = PTEN-induced kinase 1, α-SMA = alpha-smooth muscle actin.

The pathological process of alcoholic liver disease involves several components, including steatosis, apoptosis, inflammatory response and liver fibrosis. Studies have shown that LncRNAs play an important role in these areas. They can affect the expression and function of relevant genes and pathways through multiple mechanisms, including epigenetic regulation, transcriptional regulation, post-transcriptional regulation and molecular sponge effect. In this section, we will explore in detail the specific mechanisms of action of LncRNAs in different pathological processes of alcoholic liver disease and analyze their regulatory roles in steatosis, apoptosis, inflammatory response, fibrosis, etc (Fig. 1).

### 2.1. LncRNA and lipid metabolism

Hepatocellular steatosis is the initial stage in the development of ALD. Long-term consumption of large amounts of alcohol affects lipid metabolism in hepatocytes, leading to the gradual accumulation of fat and the development of alcoholic fatty liver.^[12]^ The roles and mechanisms of LncRNAs in hepatocellular steatosis are very complex, involving multiple signaling pathways and gene regulation. In ALD, the LncRNA MEG3 was found to act as a competing endogenous RNA (ceRNA) for miR-let-7c-5p and play a role in alcohol-induced liver injury. LncRNA MEG3 alters fatty acid synthesis, catabolism and storage by regulating the expression of NLRC5, activating transcription factors such as NF-κB and thus promoting hepatocyte steatosis.^[13]^ LncRNA-AIRN expression was also upregulated in the ALD model. LncRNA-AIRN leads to a decrease in mTOR protein levels by promoting the E3 ubiquitin ligase FBXW7-mediated ubiquitination of mammalian target of rapamycin (mTOR). mTOR is a key factor in the regulation of cellular autophagy, and a decrease in its protein levels activates the mitochondrial autophagy process,^[14]^ leading to the blockage of fatty acid β-oxidation^[15]^ and the accumulation of unoxidized fatty acids in cells, ultimately leading to steatosis. Mitochondrial autophagy can be reduced by decreasing the expression of LncRNA-AIRN, thereby reducing alcohol-induced liver injury and fat accumulation. Future studies should further investigate LncRNA-AIRN as a potential target for the treatment of ALD.

### 2.2. LncRNA and apoptosis

It has been found that in a mouse model of alcoholic hepatitis (AH), there is a decrease in the expression of LINC01093 and an increase in the expression of intercellular cell adhesion molecule-1 (ICAM-1). LINC01093 effectively inhibits hepatocyte apoptosis and increases hepatocyte viability by promoting proliferation.^[16]^ ICAM-1 is a transmembrane glycoprotein that is mainly expressed on the surface of endothelial cells, immune cells and certain epithelial cells.^[17]^ The intracellular portion of ICAM-1 can interact with intracellular signaling molecules and affect apoptosis-related signaling pathways. Activation of ICAM-1 can activate a variety of intracellular signaling molecules ICAM-1 activation can activate a variety of intracellular signaling molecules, such as MAPK and NF-κB, and these signaling pathways play an important role in the regulation of apoptosis. In addition, LncRNA CRNDE expression was increased in the ALD mouse model. Enrichment analysis revealed that LncRNA CRNDE was involved in extrinsic apoptotic signaling pathways through death structural domain receptors and intrinsic apoptotic signaling pathways in response to DNA damage, regeneration and regulation of connective tissue adhesion,^[18]^ suggesting that LncRNA CRNDE plays an important role in apoptotic pathways. Furthermore, the expression of LncRNA UCA1 was found to be upregulated in alcohol-induced mouse hepatocytes, and LncRNA UCA1 promotes the expression of krüppel-like factor 5 (KLF5) through competitive inhibition of miR-214.^[19]^ KLF5 is a zinc finger transcription factor belonging to the KLF family, and its can directly regulate the expression of a variety of apoptosis-related genes.^[20]^ In alcohol-induced mouse hepatocytes, LncRNA UCA1 increased the expression of KLF5, promoted the expression of pro-apoptotic genes such as p21 and Bax, and inhibited the expression of anti-apoptotic genes such as Bcl-2 by targeting miR-214. The expression level of KLF5 was significantly downregulated in alcohol-induced mouse hepatocytes after transfection with silenced UCA1 (si-UCA1).^[19]^ Therefore, UCA1 knockdown may reverse the inhibitory effect of alcohol on biological behaviors such as proliferation, migration and apoptosis of hepatocytes.

### 2.3. LncRNA and inflammatory response

The alcohol-induced hepatic inflammatory response is a key process in the progression of ALD. Studies have shown that LncRNAs play important regulatory roles in the inflammatory response. One study investigated the role of LncRNA 1700020I14Rik in the inflammatory phase of ALD and its regulatory mechanism, and found that the expression level of 1700020I14Rik was significantly increased in the liver tissue of mice in the AH model, and activated aldo-keto reductase family 1B10 (AKR1B10) extracellular signaling in the state of AH by suppressing the expression of miR-137-regulated kinase (Erk) signaling pathway,^[9]^ increasing the expression of inflammatory factors and promoting hepatic inflammatory responses. AKR1B10 belongs to the aldo-keto reductase family, which is mainly involved in lipid metabolism and oxidative stress, protecting cells from metabolites and environmental toxins by regulating cholesterol and fatty acid synthesis, vitamin A metabolism, antioxidant defence and detoxification. It is also capable of reducing a wide range of aldehyde and ketone substrates to their corresponding alcohol compounds.^[21]^ Extracellular signal-regulated kinase (Erk) is a serine/threonine kinase,^[22]^ and the Erk pathway is widely activated in inflammatory responses, promoting the expression and release of inflammatory factors such as TNF-α and IL-6.^[23]^ AKR1B10 promotes the activity of the Erk pathway by regulating lipid metabolism, indirectly contributing to inflammatory responses.

### 2.4. LncRNA and fibrosis

Liver fibrosis is an important stage in the progression of ALD to cirrhosis. LncRNAs also play a key role in the fibrosis process. Activation of hepatic stellate cells (HSCs) is an important driver of alcoholic liver fibrosis.^[24]^ HSCs are activated in response to injury or inflammatory stimuli, express α-SMA and transform into myofibroblasts. Myofibroblasts synthesize large amounts of COL1A1, leading to excessive accumulation of extracellular matrix^[25]^ and ultimately to liver fibrosis.^[26]^ LncRNA XIST is an approximately 17,000 base pair long LncRNA that is predominantly encoded on the X chromosome^[27]^ and is an important regulator of X chromosome inactivation, in addition to playing a key role in a variety of physiological and pathological processes. LncRNA XIST has been found to be highly expressed in liver tissue of ALD patients and reduces miR-29b inhibition of COL1A1 and α-SMA by competitively binding to miR-29b, which in turn promotes collagen synthesis and HSC activation and exacerbates liver fibrosis.^[28]^

LncRNA NEAT1 also plays an important role in ALD liver fibrosis. LncRNA NEAT1 promotes the expression of suppressor of cytokine signaling 2 (SOCS2) by negatively regulating the expression of miR-129-5p, a cytokine signaling inhibitory protein belonging to the SOCS family of proteins, which is known to reduce the production of pro-fibrotic cytokines by inhibiting the JAK/STAT pathway, thereby reducing the production of pro-fibrotic cytokines and slowing the progression of fibrosis.^[29]^ Inhibition of NEAT1 expression by RNA interference inhibits SOCS2 activity and slows down the liver fibrosis process in ALD.^[30]^ Therefore, NEAT1 knockdown may slow the progression of ALD to cirrhosis.

The regulatory role of LncRNA HOTAIR in a variety of liver diseases has been extensively studied, and its role in ALD liver fibrosis should not be overlooked. HOTAIR promotes the activation and proliferation of HSCs by regulating the TGF-β/Smad signaling pathway. TGF-β binds to its receptors (TGF-βRI and TGF-βRII), activating TGF-β RI, which in turn phosphorylates Smad2 and Smad3. Regulates the expression of fibrosis-related genes such as COL1A1 and α-SMA.^[31]^ In addition, HOTAIR promotes H3K27me3 modification by binding to PRC2,^[32]^ which silences negative regulator genes (e.g. Smad7) and enhances TGF-β signaling by modulating H3K27me3 modification, which promotes fibrosis-related gene expression.^[33]^

In 2018, Zhou et al found for the first time that the LncRNA Gm5091 was significantly downregulated during alcohol-induced liver fibrosis. It was found that Gm5091 had high affinity for several fibrosis-related miRNAs, including miR-27b, miR-23b and miR-24, and Gm5091 inhibited alcohol-induced HSC activation by competitively inhibiting these miRNAs, and suppressed ROS production, interleukin-1 β (IL-1 β) secretion as well as α-SMA and COL1A1 expression, and attenuated alcoholic liver fibrosis.^[34]^ Therefore, it can be suggested that LncRNA Gm5091 may be involved in the fibrotic process by regulating the activity and inflammatory response of HSCs.

The mechanism of action of LncRNAs in ALD has been widely studied and recognized (Table 1). However, there are still many potential and undiscovered mechanisms. For example, the specific functions of LncRNAs in different cell types, their roles in intercellular communication and how they integrate the regulation of multiple signaling pathways need to be further investigated. In addition, the complex network relationships between LncRNAs and other non-coding RNAs (e.g. circRNAs, piRNAs, etc) and protein-coding genes need to be explored in depth.

**Table 1:** LncRNA expression in alcoholic liver disease.

LncRNA	Expression	Mechanisms of action	Reference
LncRNA MEG3	Up	Positive regulation of NLRC5 expression as a competitive endogenous RNA for miR-let-7c-5p exacerbates alcohol-induced steatosis	^[13]^
LncRNA AIRN	Up	Interacts with mTOR proteins and inhibits their ubiquitination degradation, thereby facilitating the process of mitochondrial autophagy	^[14,15]^
LncRNA 1700020I14Rik	Up	Inhibition of miR-137 expression activates the AKR1B10-Erk signaling pathway in the AH state	^[9,21–23]^
LncRNA LINC01093	Down	Targeted inhibition of ICAM-1 and NF-κB signaling pathway increases hepatocyte viability and decreases apoptosis	^[16]^
LncRNA CRNDE	Up	Extrinsic apoptotic signaling pathways involved in death structural domain receptors, and intrinsic apoptotic signaling pathways in response to DNA damage, regeneration and regulation of adhesion-connected tissues, promote apoptosis	^[18]^
LncRNA UCA1	Up	Adsorption and reduction of miR-214 concentration to promote KLF5 expression, promote expression of pro-apoptotic genes and inhibit expression of anti-apoptotic genes	^[19,20]^
LncRNA XIST	Up	Competitive endogenous RNA as miR-29b promotes HMGB1 expression and enhances alcohol-induced HSC activation	^[27,28]^
LncRNA NEAT1	Down	Negative regulation of miR-129-5p expression promotes SOCS2 expression and slows liver fibrosis in ASH	^[29,30]^
LncRNA HOTAIR	Up	Promoting activation and proliferation of hepatic stellate cells by modulating the TGF-β/Smad signaling pathway	^[31–33]^
LncRNA Gm5091	Down	Adsorption and reduction of miR-27b/23b/24 concentrations negatively regulate alcohol-induced HSC activation and inhibit ROS production, IL-1 β secretion, and the expression of α-SMA, Desmin, and Col I to attenuate alcoholic liver fibrosis	^[34]^

Col I = type I collagen, Desmin = junctional protein, HMGB1 = high mobility group protein B1, HSC = hepatic stellate cell, ICAM-1 = intercellular cell adhesion molecule-1, IL-1β = interleukin-1β, Nrf2 = nuclear factor E2-related factor 2, ROS = reactive oxygen species, α-SMA = α-smooth muscle actin.

## 3. The potential of LncRNAs as diagnostic markers and therapeutic targets in ALD

### 3.1. Diagnostic markers and therapeutic targets

Due to the specific expression patterns of LncRNAs in certain diseases, they have been widely used as diagnostic markers and therapeutic targets in the diagnosis and treatment of diseases.^[35,36]^ Using high-throughput sequencing technology, the researchers were able to identify and quantify differentially expressed LncRNAs in ALD patients, opening up the possibility of developing new diagnostic tools. High expression levels of LncRNAs can be used as an indicator for early diagnosis of ALD, which may help to identify patients at an early stage and intervene in time to prevent disease progression. For example, LncRNA CRNDE and LncRNA XIST, which are highly expressed in ALD patients, can be used as biomarkers for early diagnosis to help identify early stage ALD patients for timely intervention.^[20,30]^ By targeting specific LncRNAs, their downstream molecular pathways can be modulated to alleviate alcohol-induced liver injury. Inhibition of TGF-βRI activation by knockdown of the LncRNA HOTAIR, which further inhibits Smad2 and Smad3 phosphorylation and leads to reduced expression of fibrosis-related genes, can attenuate liver fibrosis and improve the pathological state of ALD.^[31]^ Similarly, targeted enhancement of LINC01093 expression can reduce hepatocyte apoptosis, thereby attenuating alcohol-induced liver injury.^[22]^

### 3.2. Potential therapeutic strategies

However, the translation of LncRNAs from research objects to clinical applications still faces challenges, including the in-depth understanding of LncRNA functions, regulatory networks and disease relevance, as well as the development of effective drug delivery systems and therapeutic approaches. As technology advances and research deepens, LncRNAs are expected to become the next generation of biomarkers and therapeutic targets, bringing more precise and effective treatment options to patients.

#### 3.2.1. Regulation of ALD progression by targeting LncRNAs using RNA interference

RNA interference (RNAi) technology targets specific mRNAs using small interfering RNAs (siRNAs) or short hairpin RNAs (shRNAs) to inhibit their expression.^[37]^ Currently, many studies have shown that targeting specific LncRNAs using RNAi technology can effectively regulate their functions in disease development and progression.^[38]^ For example, targeting NEAT1 expression with siRNA can reduce the severity of ALD. In alcohol-treated mouse hepatocytes, inhibition of NEAT1 expression by specific siRNA improved liver function, reduced lipid levels, effectively suppressed inflammatory responses, prevented the occurrence of hepatocyte apoptosis and inhibited the progression of liver fibrosis.^[30]^ In addition, Akhade VS et al^[9]^ found that silencing 1700020I14Rik by shRNA could significantly reduce the expression of 1700020I14Rik in mouse liver tissue, which was accompanied by a significant decrease in serum ALT, AST, TNF-α and IL-6 levels. This suggests that the inflammatory response of ALD can be effectively attenuated by interfering with the expression of 1700020I14Rik.

Currently, the main challenge for RNAi therapy is how to deliver siRNAs efficiently and specifically to the liver. Delivery systems such as nanoparticles, liposomes and viral vectors are being developed to improve delivery efficiency and specificity.^[9]^ These systems can protect RNA molecules from degradation and ensure that siRNA or shRNA are delivered effectively and precisely to liver cells through specific targeting mechanisms, thereby reducing off-target effects and enhancing therapeutic efficiency. Among them, lipid nanoparticles (LNPs), functionalized with ligands such as N-acetylgalactosamine (GalNAc), specifically target receptors on the surface of liver cells, increasing specificity and reducing systemic side effects.^[39]^ Additionally, polymer nanoparticles and inorganic carriers (such as gold or silica nanoparticles) allow for controlled release to diseased tissues, improving drug persistence.^[40]^ Viral vectors (such as adeno-associated virus, AAV) are utilized in LncRNA-based therapies for efficiently delivering shRNA or gene editing tools to regulate specific LncRNAs.^[41]^ These vectors exhibit liver tropism and can enable long-term expression of shRNA, continuously interfering with LncRNAs associated with ALD.^[42]^ With engineered modifications, viral vectors can achieve specific targeting of liver cells, further minimizing off-target effects and side effects.^[43]^ Despite challenges such as immune responses, viral vectors remain important tools in LncRNA-targeted therapies.^[44]^ In addition, RNAi can have off-target effects and affect the expression of non-target genes.^[45]^ Optimizing siRNA design and selecting targets with high specificity can reduce this risk. Individualized design of RNAi therapies based on patient-specific genetic profiles and disease progression can improve therapeutic efficacy and reduce side effects.^[46]^

Overall, RNA interference technology holds great promise for the study and treatment of ALD. With continued advancement and optimization of the technology, RNAi is expected to become an effective treatment for these diseases.

#### 3.2.2. Potential of small molecule drugs targeting LncRNAs in ALD treatment

Small molecule drugs are now widely used in the clinic.^[47,48]^ Small molecule drugs regulate biological functions by specifically binding to target molecules. The development of small molecule drugs to modulate the expression and function of LncRNAs may be another potential strategy for the treatment of ALD. Targeting ALD-related LncRNAs and screening for small molecules that can bind to specific LncRNAs could affect their stability or interaction with other molecules, block their regulatory effects on pathways associated with ALD occurrence, and intervene in the progression of ALD. The small molecule antioxidant N-acetylcysteine attenuates alcohol-induced liver injury by scavenging ROS and improving antioxidant capacity.^[49]^ The NOX1/4 inhibitor GKT137831 acts as a dual inhibitor of NOX1 and NOX4, which protects hepatocytes from injury, reduces serum aminotransferase levels and improves hepatic steatosis by reducing ROS and inflammatory mediators.^[50]^ However, the diversity and complexity of LncRNA structures make the design and screening of small molecule drugs challenging. Overall, the use of small molecule drugs in the treatment of ALD is promising, but a large number of clinical trials are still needed to verify their efficacy and safety.

#### 3.2.3. CRISPR/Cas9-based gene editing of LncRNAs for ALD research and treatment

Gene editing technologies, such as the CRISPR/Cas9 system, have shown great potential in disease research and treatment.^[51,52]^ It provides a method to precisely regulate LncRNA expression at the gene level. The knockdown or knock-in of specific LncRNA genes is achieved by designing guide RNAs (gRNAs) to direct the Cas9 nuclease to specific genomic locations.^[53]^ This approach can permanently alter the expression pattern of LncRNAs, which is important for studying the role of LncRNAs in ALD and their potential as therapeutic targets. However, the application of gene editing technology still faces clinical translational challenges, including issues of targeting, editing efficiency and potential nonspecific editing.

## 4. Outlook

In future research, the role and therapeutic potential of LncRNAs in ALD will continue to be a crucial area of exploration. Given the diverse regulatory functions of LncRNAs in liver injury, lipid metabolism disorders, inflammatory responses, and fibrosis progression, in-depth investigation of these molecular mechanisms may reveal new therapeutic targets. With the ongoing advancements in RNAi and gene editing technologies, personalized therapeutic approaches targeting LncRNAs are likely to become achievable. However, several challenges remain, including improving treatment specificity, minimizing off-target effects, and ensuring long-term safety. Additionally, personalized interventions based on patient-specific genetic profiles and disease stages will be key to enhancing efficacy and reducing side effects in future ALD treatments. In conclusion, the role of LncRNAs in ALD and their therapeutic potential are highly promising, and future research is expected to drive this field from basic biological research toward clinical applications, offering new therapeutic options for ALD patients.

## 5. In summary

LncRNAs play an important role in the development and progression of ALD and are involved in several pathological processes, including lipid metabolism, apoptosis, inflammatory response and fibrosis. Although studies have demonstrated the important role of LncRNAs in ALD, relatively few studies have been conducted in this area. Most studies have focused on LncRNA-miRNA interaction networks, while their role in the development of ALD is poorly understood. In addition, the highly heterogeneous nature of ALD pathogenesis, which may result in different LncRNA expression profiles for different individuals, drinking patterns and genetic backgrounds, remains a challenge to explore the regulation of LncRNA expression or function for the treatment of ALD.

The potential of LncRNAs as therapeutic targets is enormous, but no therapeutic strategy against LncRNAs has yet been applied in the clinic. Further studies are needed to determine its feasibility and safety in the treatment of ALD. Given its critical role in ALD, it is expected to be a new diagnostic marker and therapeutic target. Future studies should further elucidate the specific mechanism of action of LncRNA, develop effective LncRNA-targeted therapeutic strategies to control the onset and progression of ALD, and improve the clinical prognosis of ALD.

## Author contributions

**Conceptualization:** Rongqi Wang, Yuemin Nan, Lingbo Kong.

**Methodology:** Rongqi Wang.

**Supervision:** Lingbo Kong.

**Writing – original draft:** Lin Zhang, Lingbo Kong.
